# Associations between Abnormal Eating Styles and Irritable Bowel Syndrome: A Cross-Sectional Study among Medical School Students

**DOI:** 10.3390/nu14142828

**Published:** 2022-07-09

**Authors:** Wenhan Jia, Hong Liang, Lining Wang, Ming Sun, Xili Xie, Jie Gao, Linxian Li, Xiao Tang, Yanan Ma

**Affiliations:** 1 Department of Biostatistics and Epidemiology, School of Public Health, China Medical University, Shenyang 110122, China; 2020120352@stu.cmu.edu.cn (W.J.); hliang@cmu.edu.cn (H.L.); lnwang@cmu.edu.cn (L.W.); msun97@cmu.edu.cn (M.S.); xlxie@cmu.edu.cn (X.X.); jiegao@cmu.edu.cn (J.G.); 2 Public Administration School, Renmin University of China, No. 59 Zhongguancun Street, Haidian District, Beijing 100872, China; lilinxian1991@163.com; 3 School of Public Health, Dalian Medical University, No. 9 South Road, Lvshun District, Dalian 116044, China; xtang@cmu.edu.cn

**Keywords:** irritable bowel syndrome, eating style, emotional eating, restraint eating, external eating

## Abstract

(1) Background: Few studies have investigated the association between eating styles and IBS. This study aimed to explore the association between abnormal eating styles and irritable bowel syndrome (IBS). (2) Methods: This cross-sectional study investigated students in China Medical University and Shenyang Medical College. Eating styles were evaluated by the Dutch Eating Behavior Questionnaire (DEBQ), and IBS was diagnosed according to Rome III criteria. Logistic regression was used to estimate odds ratios (ORs) and 95% confidence intervals (CIs). (3) Results: A total of 335 students were diagnosed with IBS. Students with the total scores in tertile 2 and 3 had 1.29 times and 2.75 times higher risk of IBS than students with the total scores in tertile 1, respectively. Simultaneously, the risk of IBS in the tertile 3 of external eating, emotional eating, and restraint eating trends was 3.87 times, 2.71 times, and 3.82 times higher than that of tertile 1, respectively. (4) Conclusions: this study showed that a high score in both total eating styles and each eating style was associated with the odds of having IBS and suggested that the psychological factors behind eating styles may play a critical role in controlling the IBS.

## 1. Introduction

Irritable bowel syndrome (IBS) is a disorder of gut brain interaction (DGBI), characterized by chronic abdominal pain and altered bowel habits, in the absence of any other disease [1]. As a common gastrointestinal disease, about 10% to 15% of the world’s population suffers from IBS, and the prevalence in Asia ranges from 6.8% to 33.3% [2,3]. Leading to uncontrollable intestinal symptoms, such as constipation and diarrhea, IBS has a marked adverse impact on patients’ quality of life, causing appetite decrease, psychomotor retardation, dysphoria, depression, and other serious issues [4,5,6,7]. Meanwhile, health care and medication costs remain higher and keep increasing during IBS remission than in other DGBI, which places a heavy burden on the healthcare system [8,9]. Moreover, IBS increases patient absence from work, which places stress on a patient’s personal financial life [9,10]. Therefore, it is essential to study the risk factors associated with IBS to develop prevention and control strategies. Early life stressors (abuse and psychosocial stressors), antibiotics, enteric infection, and dietary factors have been considered environmental contributors to IBS symptoms worldwide [11]. Thorough identification of modifiable risk factors is significant in alleviating IBS symptoms.

Eating style is a psychology-related concept known as ‘one’s cues to eat’, including emotional eating, restraint eating, and external eating, which could determine the functioning of both adults and children [12]. In simple terms, emotional eating is associated with the tendency to eat due to negative emotions; similarly, external eating implies eating stimulated by environmental factors, such as the aroma and appearance of delicious food; restraint eating represents the act of reducing caloric intake for weight control [13,14,15]. According to reports, these eating styles could negatively affect human health, connecting with depression, overeating, and obesity [16,17,18]. Many researchers have proved the association between diet-related risk factors and IBS, such as binge eating, high-calorie food intake, and irregular eating rhythms [19,20,21]. However, few studies have investigated the association between eating styles and IBS. Recently, the prevalence of abnormal eating styles and IBS among Chinese college students remains high [22,23]. Thus, evaluating the relationship between them can control IBS symptoms at both the psychological and behavioral levels and provide further insights into the harm of the three eating styles.

Accordingly, we investigated eating styles and IBS prevalence in a representative sample of Chinese medical students and examined the role of the three different eating styles in IBS symptoms. We hypothesize a positive relationship between abnormal eating styles and the odds of having IBS.

## 2. Materials and Methods

### 2.1. Study Design and Sample Collection

This cross-sectional study investigates the relationship between IBS and eating styles, conducted in June 2021 and finished by students at China Medical University and Shenyang Medical College. A random sampling method was used to select representative first-year students to senior students as the survey objects and obtained the information needed for research through an online questionnaire. A total of 2739 students who signed voluntary consent participated in the study and made up the final sample, after excluding participants who met the exclusion criteria. The Ethics Review Board approved our study at China Medical University. The inclusion criteria are (1) students who volunteered and signed informed consent; (2) first-year students to senior students in China Medical University and Shenyang Medical College. Moreover, the exclusion criteria for excluding participants with other conditions that have similar symptoms to IBS are as follows: (1) participants who suffer from other primary gastrointestinal diseases that can better explain their gastrointestinal symptoms; (2) participants who have been diagnosed with malignancies other than basal cell carcinoma of the skin or squamous cell carcinoma within the past 5 years; (3) participants with unstable extraintestinal disease; (4) participants who have a fever, gastrointestinal bleeding, weight loss, anemia, abdominal mass, and other “alarming” symptoms or signs that are not due to IBS; (5) participants with a history of alcohol or drug abuse; (6) female participants with dysmenorrhea during menstruation [24,25].

### 2.2. Assessment of IBS

We diagnosed IBS by a self-report questionnaire developed according to the Rome III criteria [26]. IBS was defined as having recurrent abdominal pain or discomfort for at least 3 days per month in the past 3 months and being accompanied by two or more of the following symptoms: (1) symptoms that improved after defecation; (2) changes in defecation frequency; (3) changes in stool properties [27].

### 2.3. Assessment of Eating Styles

Eating styles were assessed by the Dutch Eating Behavior Questionnaire (DEBQ), an international tool widely used to assess eating styles, which determines the severity of the three eating styles by assessing individuals’ thoughts, motivations, and feelings about food [28]. DEBQ has a total of 33 items, including 3 subscales, namely the restraint eating subscale (containing 10 items), the emotional eating subscale (containing 13 items), and the external eating subscale (containing 10 items). Each item is scored on a scale of 1–5, and the scores for all items in each subscale are added together, with a higher total indicating a greater propensity to choose the corresponding eating style. Moreover, the total score is obtained by adding the scores for the three eating styles. The emotional eating subscale and the extrinsic eating subscale reflect the impulsive eating style, and restraint eating reflects inhibited eating style. We used the Chinese version of DEBQ to assess eating styles, which has been shown to be a psychometrically reliable and valid instrument with potential for assessment [29,30].

### 2.4. Assessment of Covariates

A self-administered questionnaire was used to obtain information about the potential covariates, including age (years), BMI (body mass index) (kg/m^2^), gender (male, female), monthly living expenses (CNY) (≤1200, >1200), major (clinical medicine, others), smoking (no, yes), drinking (no, yes) and physical activity (inactive, partially active, active). BMI was calculated by dividing self-reported weight (kg) by self-reported height (m) squared. In addition, we used an established reference value of BMI = 27 kg/m^2^ as a cut-off point for defining obesity according to international standards. The International Physical Activity Questionnaire-Short Questionnaire (IPAQ-S) evaluated physical activity status and was divided into the following three ranks: inactive, partially active, and active [31]. To distinguish clinical students from students of other majors, we divided majors into “clinical medicine” and “others”; “others” includes anesthesiology, nursing, pharmacy, public health, forensic science, and other medical professions.

### 2.5. Statistical Analysis

We expressed continuous variables with means ± standard deviation (SD) and categorical variables with percentages. To examine the effect of different eating style levels on IBS, we divided the total score and score for the three eating styles into tertiles. Tertile 1 deserved the lowest score, and tertile 3 was the group with the highest score. We used variance analysis (ANOVA) for continuous variables and chi-square tests for categorical variables to compare general characteristics among the eating style levels. Binary logistic regressions were used to estimate odds ratios (ORs) and 95% confidence intervals (CIs), and three models were calculated. Firstly, age was adjusted in model I. Second, age, gender, and BMI were adjusted in model II. Third, in model III, age, BMI, gender, monthly living expenses, major, smoking, drinking, and physical activity were adjusted. In addition to examining the relationship between the total score for eating styles and IBS, we also analyzed the relationship between each of the three eating styles (emotional eating, external eating, and restraint eating) and IBS separately and adjusted each of the three eating styles in multivariable-adjusted models. Moreover, to explore whether the association between eating styles and IBS is consistent among the participants with different general characteristics, we conducted a stratified analysis according to different general characteristics. The two-tailed test indicated significance at a statistical level of 0.05. All statistical analyses were performed by IBM SPSS Statistics 21.0 software (IBM, Asia Analytics Shanghai, Shanghai, China).

## 3. Results

### 3.1. Baseline Characteristics

Initially, 6078 students met the inclusion criteria, and after the exclusion, 2739 students constituted the final study subjects. A total of 335 students were diagnosed with irritable bowel syndrome (221 males and 114 females), with a prevalence of 12.23%. General characteristics among the study participants across tertiles of eating styles are provided in Table 1. Students with the highest eating styles (the third tertile) were more likely to be male, receive monthly living expenses less than CNY 1200, suffer from obesity, with a major other than clinical medicine, smoking, drinking, and be inactive in physical activity. In addition, we compared the difference in general characteristics among study participants across tertiles of emotional eating, external eating, and restraint eating separately and obtained the same results as the total score (Tables S1–S3). According to the results of the univariate analysis in Table 2, males, monthly living expenses less than CNY 1200, non-clinical professionals, smoking, drinking, and inactive physical activity are all risk factors for IBS (*p* < 0.05).

### 3.2. Association between the Total Score for Eating Styles and IBS

We analyzed the association between the total score for eating styles and IBS in different models (Table 3). In the model I adjusted for age, compared with those with the total scores in tertile 1, students with the total scores in tertile 2 had a 2.89 times risk of having IBS (OR: 2.89; 95% CI: 1.90–4.39), and students with the total scores in tertile 3 had an 8.64 times risk of having IBS (OR: 8.64; 95% CI: 5.83–12.81). A similar result was obtained in Model II as in Model I, and even after controlling for many potential confounders in model III, abnormal eating styles were still associated with a greater risk of IBS. Students with the total scores in tertile 2 and 3 had 1.29 times (OR: 2.29; 95% CI: 1.48–3.54) and 2.75 times (OR: 3.75; 95% CI: 2.40–5.86) higher risk of IBS than students with the total scores in tertile 1, respectively.

### 3.3. Association between Different Eating Styles and IBS

We analyzed the association between IBS and the three eating styles in Table 4 separately. As can be observed, the more pronounced the tendency of the three eating styles, the greater the possibility of suffering from IBS, which was very significant in models I and II. The association was still significant for restraint eating and external eating after adjusting each one for different eating styles in model III. For emotional eating, it became non-significant in model III.

### 3.4. Association between the Total Score for Eating Styles and IBS in Stratified Analyses

The significant associations between eating styles and IBS risk remained in each stratified analysis for potential confounders (age, BMI, gender, monthly living expenses, major, smoking, drinking), except for physical activity. In the stratified analysis of physical activity, a significant relationship between eating styles and IBS probabilities was observed only in active physical activity and inactive physical activity (Table 5). In addition, in the interaction analysis, we found a significant interaction between each potential confounder and eating styles. We also performed a stratified analysis of the three eating styles separately and obtained similar results for the overall eating style. However, as for emotional eating, there was no significant association of eating styles with IBS that was observed in different genders or clinical specialty for the other two eating styles (Tables S4–S6).

## 4. Discussion

This cross-sectional study showed that, among 2739 Chinese medical students, the total eating styles score and the three eating styles were associated with the odds of having IBS. According to the study results, the risk of IBS of the students with the total scores in tertile 3 was 3.75 times that in tertile 1. Furthermore, the risk of IBS in the tertile 3 of external eating, emotional eating, and restraint eating trends was 3.87 times, 2.71 times, and 3.82 times higher than that of tertile 1, respectively.

In previous studies on IBS and diet-related factors, most explored the impact of dietary content and quantity on IBS. For example, in the Swedish Twin Study of Adults, there was a significant positive association between binge eating and IBS symptoms among 23,821 adults [32]. An observational study that explored the relationship between IBS and alcohol consumption found a stronger association between binge drinking and the next day’s gastrointestinal symptoms than drinking [33]. A low-FODMAP diet is characterized by a limited intake of foods containing highly fermentable oligosaccharides, disaccharides, and monosaccharides and polyols (FODMAPs), such as fruits, vegetables, legumes and cereals, honey, milk, and dairy products, which have been shown to have a beneficial effect on IBS-related symptoms repeatedly [34,35]. In addition, the connection between unhealthy foods and IBS was also testified. A prospective dietary intervention study for 105 adult IBS patients in Sweden found that IBS patients who ate too much fast and processed food, cereals, and sweets/soft drinks were more likely to suffer from aggravated gastrointestinal symptoms [36]. In addition, many IBS-related dietary guidelines showed that reducing the intake of caffeine, alcohol, spicy foods, fat, and milk, and increasing the intake of fiber-rich foods should be the first line of treatment for IBS [19].

Our study compared to the above studies testified to the relationship between eating styles and IBS. Eating styles are related to patients’ eating behaviors and the psychological factors that drive those eating behaviors. Firstly, many researchers have suggested that patients with emotional eating cannot distinguish between hunger and other negative emotions [37] or choose an inappropriate method to deal with their negative emotions [38]. Thus, they eat large amounts of food, including highly unhealthy food, to regulate distress, anxiety, stress, and other psychological problems [39]. Secondly, patients with external eating may be more responsive to external food cues and less sensitive to internal hunger and satiety signals, leading to binge drinking and binge eating when exposed to food-filled environments [40]. Thirdly, patients with restraint eating often set strict rules to reduce food intake at first. As time passes, the body may not be able to distinguish food shortage from self-imposed food restriction and acts as if in starvation mode, resulting in more hunger and greater appetite than before [15]. On the other hand, such strict rules will become increasingly difficult to maintain, and the inevitable breaking may induce overeating [41]. Many researchers have proposed that negative emotions can further weaken the control of diet in patients with external eating and restraint eating, increasing their need for overeating [37,40,42]. In addition, numerous studies have proven that those three eating styles can lead to binge eating [13,43,44,45,46], which is associated with IBS. To sum up, by comparing articles that only investigated the relationship between dietary content and IBS, we may have uncovered the possible psychological problems behind the eating behaviors of IBS patients and established a path from psychological factors to eating behaviors to IBS. However, since many studies recommend that IBS patients should choose low-stimulating foods and avoid overeating [19,20,21], most IBS patients also believe that their symptoms are closely related to food [47], not to mention that IBS symptoms can have a serious negative impact on quality of life [48]; thus, IBS patients will spare no effort to control their diet. As a result, people with IBS may cause restraint eating because of the restricted diet. Similarly, IBS patients may not overeat when faced with negative emotions and external food stimulation, so it is less likely to lead to emotional eating and external eating. Therefore, we will build cohort studies in the future to find evidence for the relationship between IBS and diet.

We conjecture that there may be two mechanisms for this association. First, eating styles and IBS are related to psychological factors. It is well known that psychological factors are significant contributors to IBS. Stress or negative emotions may cause gastrointestinal symptoms in patients under the effect of the brain–gut axis [49,50]. On the other hand, eating style describes eating behaviors as psychological drivers. For example, emotional eating represents eating behaviors that deal with negative emotions through eating [18,39,51]. Likewise, restraint and external eating have also been highly connected with anxiety and depressive symptoms [52,53,54,55]. Above all, negative emotions are likely to be a key variable affecting emotional eating and IBS, which needs to be further verified in future studies. The second potential mechanism is that abnormal dietary patterns can lead to a high intake of unhealthy foods. The resulting gastrointestinal intolerance and sensitivities to unhealthy foods contribute to developing IBS symptoms [20].

The limitations of this study are as follows. First, this study was conducted on Chinese medical students, so the results may be neither generalizable to other countries nor necessarily applicable to different age groups. Second, this study is cross-sectional, which means it cannot establish a causal link between IBS and eating style. Third, we only used DEBQ to assess eating styles in this study. The Three-Factor Eating Questionnaire (TFEQ-R21) can also estimate emotional eating. The different assessment instruments may lead to a different association between IBS and eating styles. Forth, there was a selection bias in our study. Due to the higher prevalence of males in the students who agreed to participate in the study, only one-third of the three-hundred and thirty-five students diagnosed with IBS were female, in contrast to the epidemic expectations. Thus, we performed a stratified analysis (Table 5), and the results showed that eating styles were strongly associated with IBS in both male (tertile 2, OR: 3.09; 95% CI: 1.64–5.84) (tertile 3, OR: 8.50; 95% CI: 4.70–15.38) and female (tertile 2, OR: 1.73; 95% CI: 0.96–3.12) (tertile 3, OR: 2.41; 95% CI: 1.26–4.59) participants; we will demonstrate the relationship between psychological and dietary problems in IBS and sex in future research. Fifth, IBS criteria were self-reported by the participants, which would result in information bias. The related problems in the questionnaire are strictly formulated according to the criteria to improve the accuracy of IBS diagnosis. Furthermore, many researchers use self-reported questionnaires to evaluate IBS, demonstrating the high reliability of the diagnostic results of this method [56,57,58]. Sixth, we cannot identify people with more than one eating style now, but we will work to overcome this challenge in the next step of our research.

## 5. Conclusions

This study conducted in a Chinese medical school revealed that both the total eating styles score and each eating style score were associated with the odds of having IBS. The higher the score for eating styles, the higher risk of developing IBS. Our findings showed that controlling the occurrence of IBS requires attention to the daily dietary intake and further consideration of the possible psychological problems behind the eating behaviors, which is of great significance for alleviating and treating IBS in the future.

## Figures and Tables

**Table 1:** Characteristics of participants according to tertiles of eating styles (*n* = 2739).

Characteristic	The Total Score for Eating Styles			
	Tertile 1	Tertile 2	Tertile 3	*p*-Value
*N*	904	923	912	
Age (years)	21.35 ± 2.58	21.54 ± 2.65	22.19 ± 2.94	<0.001
BMI (kg/m^2^)	22.76 ± 6.57	22.89 ± 6.61	23.27 ± 7.20	0.318
Gender				<0.001
Male	590 (34.34)	532 (30.97)	596 (34.69)	
Female	314 (34.73)	391 (42.36)	316 (34.65)	
Monthly living expenses (CNY)				<0.001
≤1200	418 (46.24)	421 (45.61)	542 (59.43)	
>1200	486 (53.76)	502 (54.39)	370 (40.57)	
Major				<0.001
Clinical medicine	353 (39.05)	337 (36.51)	218 (23.90)	
Others	551 (60.95)	586 (63.49)	694 (76.10)	
Obesity				0.106
No	754 (87.98)	768 (88.89)	591 (85.40)	
Yes	103 (12.02)	96 (11.11)	101 (14.60)	
Smoking				<0.001
No	756 (83.63)	696 (75.41)	353 (38.71)	
Yes	148 (16.37)	227 (24.59)	559 (61.29)	
Drinking				<0.001
No	668 (73.89)	531 (57.53)	320 (35.09)	
Yes	236 (26.11)	392 (42.47)	592 (64.91)	
Physical activity				<0.001
Inactive	503 (59.88)	519 (59.79)	713 (85.70)	
Partially active	182 (21.67)	199 (22.93)	52 (6.25)	
Active	155 (18.45)	150 (17.28)	67 (8.05)	

Values were means (standard deviation) or *n* (percentages), and values of polytomous variables may not sum to 100% because of rounding. Abbreviations: BMI, body mass index.

**Table 2:** The association between characteristics of participants and irritable bowel syndrome by univariate analysis (*n* = 2739).

Covariate	OR (95% CI)	*p*
Age (years)	0.96 (0.91,1.00)	0.051
BMI (kg/m^2^)	0.99 (0.97,1.01)	0.335
Gender		0.190
Male	1.00	
Female	0.85 (0.67,1.08)	
Monthly living expenses (CNY)		0.061
≤1200	1.00	
>1200	0.80 (0.64,1.01)	
Major		<0.001
Clinical medicine	1.00	
Others	2.03 (1.54,2.67)	
Obesity		0.451
No	1.00	
Yes	0.85 (0.55,1.31)	
Smoking		<0.001
No	1.00	
Yes	3.18 (2.52,4.02)	
Drinking		<0.001
No	1.00	
Yes	2.85 (2.24,3.63)	
Physical activity		<0.001
Inactive	1.00	
Partially active	0.43 (0.29,0.66)	
Active	0.49 (0.34,0.72)	

Abbreviations: CI, confidence interval; OR, odds ratio.

**Table 3:** Association between the total score for eating styles and irritable bowel syndrome in different models (*n* = 2739).

Exposure	Model I	Model II	Model III
	OR (95% CI)	OR (95% CI)	OR (95% CI)
The total score for eating styles			
Tertile 1	1.00	1.00	1.00
Tertile 2	2.89 (1.90,4.39)	2.62 (1.70,4.01)	2.29 (1.48,3.54)
Tertile 3	8.64 (5.83,12.81)	5.17 (3.39,7.87)	3.75 (2.40,5.86)
*p* for trend	<0.001	<0.001	<0.001

Model I was adjusted for age. Model II was adjusted for age, gender and obesity. Model III was adjusted for age, gender, obesity, monthly living expenses, major, smoking, drinking, and physical activity.

**Table 4:** Association between different eating styles and irritable bowel syndrome in different models (*n* = 2739).

Exposure	Model I	Model II	Model III
	OR (95% CI)	OR (95% CI)	OR (95% CI)
Emotional eating			
Tertile 1	1.00	1.00	1.00
Tertile 2	1.73 (1.15,2.60)	1.52 (1.00,2.30)	0.83 (0.51,1.34)
Tertile 3	3.81 (2.57,5.64)	2.71 (1.77,4.14)	0.93 (0.51,1.69)
*p* for trend	<0.001	<0.001	0.714
External eating			
Tertile 1	1.00	1.00	1.00
Tertile 2	2.87 (1.83,4.49)	2.68 (1.70,4.22)	2.00 (1.18,3.37)
Tertile 3	5.09 (3.31,7.81)	3.87 (2.48,6.03)	2.57 (1.43,4.62)
*p* for trend	<0.001	<0.001	0.006
Restraint eating			
Tertile 1	1.00	1.00	1.00
Tertile 2	2.81 (1.82,4.35)	2.61 (1.67,4.09)	2.04 (1.21,3.44)
Tertile 3	5.02 (3.27,7.71)	3.82 (2.42,6.03)	2.19 (1.20,4.00)
*p* for trend	<0.001	<0.001	0.019

Model I was adjusted for age. Model II was adjusted for age, gender and obesity. Model III was adjusted for age, gender, obesity, monthly living expenses, major, smoking, drinking, and physical activity. Of note, Emotional eating, external eating, and restraint eating variables examined in this table were adjusted for one another.

**Table 5:** Stratified analyses for association between the total score for eating styles and irritable bowel syndrome (*n* = 2739).

Exposure	The Total Score for Eating Styles			*p* for Interaction
	Tertile 1	Tertile 2	Tertile 3	
Gender				<0.001
Male (*N* = 1718)	1.00	3.09 (1.64,5.84)	8.50 (4.70,15.38)	
Female (*N* = 1021)	1.00	1.73 (0.96,3.12)	2.41 (1.26,4.59)	
Monthly living expenses (CNY)				<0.001
≤1200 (*N* = 1381)	1.00	2.70 (1.41,5.16)	7.03 (3.81,12.96)	
>1200 (*N* = 1358)	1.00	2.23 (1.25,3.96)	4.04 (2.25,7.26)	
Major				<0.001
Clinical medicine (*N* = 908)	1.00	1.63 (0.83,3.20)	1.98 (0.95,4.13)	
Others (*N* = 1831)	1.00	3.51 (1.99,6.17)	8.59 (4.97,14.86)	
Smoking				<0.001
No (*N* = 1805)	1.00	2.43 (1.49,3.95)	3.74 (2.21,6.33)	
Yes (*N* = 934)	1.00	2.65 (1.08,6.52)	8.11 (3.61,18.18)	
Drinking				<0.001
No (*N* = 1519)	1.00	2.72 (1.55,4.77)	5.21 (2.92,9.32)	
Yes (*N* = 1220)	1.00	2.22 (1.15,4.31)	5.14 (2.76,9.56)	
Physical activity				<0.001
Inactive (*N* = 1735)	1.00	3.14 (1.77,5.56)	6.89 (4.02,11.82)	
Partially active (*N* = 433)	1.00	1.65 (0.72,3.77)	1.02 (0.26,4.01)	
Active (*N* = 372)	1.00	1.60 (0.51,5.07)	4.51 (1.40,14.52)	

Adjusted for age, gender, monthly living expenses, major, smoking, drinking, and physical activity. Of note, variables examined in this table were not adjusted.

## Data Availability

The data presented in this study are available upon request from the corresponding author. The data are not publicly available due to ethical restrictions of the protocol mentioned in our approved local ethical application that data will not be available to the general public.

## Supplementary Materials

The following are available online at https://www.mdpi.com/article/10.3390/nu14142828/s1, Table S1. Characteristics of participants according to tertiles of external eating; Table S2. Characteristics of participants according to tertiles of restraint eating; Table S3. Characteristics of participants according to tertiles of emotional eating; Table S4. Stratified analyses for association between emotional eating and irritable bowel syndrome; Table S5. Stratified analyses for association between external eating and irritable bowel syndrome; Table S6. Stratified analyses for association between restraint eating and irritable bowel syndrome.

## Abbreviations

IBS	Irritable bowel syndrome
DGBI	Disorder of gut brain interaction
DEBQ	The Dutch Eating Behavior Questionnaire
BMI	Body mass index
IPAQ-S	Physical Activity Questionnaire-Short Questionnaire
SD	Standard deviation
ANOVA	Analysis of variance
OR	Odds ratio
CI	Confidence interval
