# Supplementary material for: Associations between Abnormal Eating Styles and Irritable Bowel Syndrome: A Cross-Sectional Study among Medical School Students
Source: Nutrients. 2022 Jul 9;14(14):2828. doi: 10.3390/nu14142828 (PMC9319336; doi:10.3390/nu14142828)
Supplement: Supplementary file 1 [file nutrients-14-02828-s001.zip › nutrients-1738295-supplementary.pdf]

nutrients

MDPI

Table S1. Characteristics of participants according to tertiles of external eating.

Characteristic	External Eating		
	Tertile 1	Tertile 2	Tertile 3
**N**	893	857	989
**Age (years)**	21.59 ± 2.68	21.48 ± 2.65	21.951 ± 2.86
**BMI (kg/m²)**	22.97 ± 6.63	22.84 ± 6.63	23.03 ± 7.06
**Gender**			
Male	597 (66.85)	505 (58.93)	616 (62.29)
Female	296 (33.15)	352 (41.07)	373 (37.71)
**Monthly living expenses (yuan)**			
≤1200	424 (47.48)	406 (47.37)	551 (55.71)
＞1200	469 (52.52)	451 (52.63)	438 (44.29)
**Major**			
Clinical Medicine	328 (36.73)	306 (35.71)	274 (27.70)
Others	565 (63.27)	551 (64.29)	715 (72.30)
**Obesity**			
No	739 (87.46)	705 (88.46)	669 (86.77)
Yes	106 (12.54)	92 (11.54)	102 (13.23)
**Smoking**			
No	719 (80.52)	628 (73.28)	458 (46.31)
Yes	174 (19.48)	229 (26.72)	531 (53.69)
**Drinking**			
No	640 (71.67)	493 (57.53)	386 (39.03)
Yes	253 (28.33)	364 (42.47)	603 (60.97)
**Physical activity**			
Inactive	543 (65.11)	477 (60.15)	715 (78.31)
Partially active	147 (17.63)	187 (23.58)	99 (10.84)
Active	144 (17.27)	129 (16.27)	99 (10.84)

Values were means (standard deviation) or n (percentages), and values of polytomous variables may not sum to 100% because of rounding.

Table S2. Characteristics of participants according to tertiles of restraint eating.

Characteristic	Restraint Eating		
	Tertile 1	Tertile 2	Tertile 3
**N**	883	938	918
**Age (years)**	21.42 ± 2.64	21.66 ± 2.68	21.98 ± 2.88
**BMI (kg/m²)**	22.60 ± 6.61	23.20 ± 6.74	23.06 ± 6.99
**Gender**			
Male	589 (66.70)	548 (58.42)	581 (63.29)
Female	294 (33.30)	390 (41.58)	337 (36.71)
**Monthly living expenses (yuan)**			
≤1200	413 (46.77)	448 (47.76)	520 (56.64)
＞1200	470 (53.23)	490 (52.24)	398 (43.36)
**Major**			
Clinical Medicine	351 (39.75)	331 (35.29)	226 (24.62)
Others	532 (60.25)	607 (64.71)	692 (75.38)
**Obesity**			
No	735 (88.34)	766 (87.64)	612 (86.56)
Yes	97 (11.66)	108 (12.36)	95 (13.44)

**Smoking**			
No	738 (83.58)	685 (73.03)	382 (41.61)
Yes	145 (16.42)	253 (26.97)	536 (58.39)
**Drinking**			
No	644 (72.93)	542 (57.78)	333 (36.27)
Yes	239 (27.07)	396 (42.22)	585 (63.73)
**Physical activity**			
Inactive	507 (61.45)	541 (61.97)	687 (81.59)
Partially active	168 (20.36)	190 (21.76)	75 (8.91)
Active	150 (18.18)	142 (16.27)	80 (9.50)

Values were means (standard deviation) or n (percentages), and values of polytomous variables may not sum to 100% because of rounding.

Table S3. Characteristics of participants according to tertiles of emotional eating.

**Characteristic**	**Emotional Eating**		
	**Tertile 1**	**Tertile 2**	**Tertile 3**
**N**	842	959	938
**Age (years)**	21.31 ± 2.56	21.61 ± 2.64	22.13 ± 2.95
**BMI (kg/m²)**	22.77 ± 6.62	22.96 ± 6.61	23.13 ± 7.13
**Gender**			
Male	532 (63.18)	566 (59.02)	620 (66.10)
Female	310 (36.82)	393 (40.98)	318 (33.90)
**Monthly living expenses (yuan)**			
≤1200	363 (43.11)	469 (48.91)	549 (58.53)
＞1200	479 (56.89)	490 (51.09)	389 (41.47)
**Major**			
Clinical Medicine	337 (40.02)	343 (35.77)	228 (24.31)
Others	505 (59.98)	616 (64.23)	710 (75.69)
**Obesity**			
No	704 (87.89)	795 (88.63)	614 (85.87)
Yes	97 (12.11)	102 (11.37)	101 (14.13)
**Smoking**			
No	713 (84.68)	721 (75.18)	371 (39.55)
Yes	129 (15.32)	238 (24.82)	567 (60.45)
**Drinking**			
No	618 (73.40)	572 (59.65)	329 (35.07)
Yes	224 (26.60)	387 (40.35)	609 (61.93)
**Physical activity**			
Inactive	427 (54.39)	572 (63.63)	736 (85.98)
Partially active	191 (24.33)	187 (20.80)	55 (6.43)
Active	167 (21.27)	140 (15.57)	65 (7.59)

Values were means (standard deviation) or n (percentages), and values of polytomous variables may not sum to 100% because of rounding.

Table S4. Stratified analyses for association between emotional eating and irritable bowel syndrome.

Exposure	Emotional Eating			*p* for Interaction
	Tertile 1	Tertile 2	Tertile 3	
**Gender**				＜0.001
Male (N = 1718)	1.00	3.20 (1.61,6.38)	8.82 (4.57,17.00)	
Female (N = 1021)	1.00	0.91 (0.53,1.55)	1.45 (0.80,2.61)	
**Monthly living expenses (yuan)**				＜0.001
≤1200 (N = 1381)	1.00	3.71 (1.81,7.58)	7.67 (3.79,15.50)	
＞1200 (N = 1358)	1.00	0.91 (0.53,1.56)	2.56 (1.53,4.26)	
**Major**				＜0.001
Clinical Medicine (N = 908)	1.00	1.13 (0.59,2.17)	1.36 (0.66,2.79)	
Others (N = 1831)	1.00	2.18 (1.28,3.71)	6.06 (3.63,10.11)	
**Smoking**				＜0.001
No (N = 1805)	1.00	1.35 (0.86,2.13)	2.52 (1.55,4.11)	
Yes (N = 934)	1.00	3.32 (1.21,9.12)	9.15 (3.57,23.48)	
**Drinking**				＜0.001
No (N = 1519)	1.00	1.61 (0.95,2.71)	3.19 (1.85,5.50)	
Yes (N = 1220)	1.00	1.64 (0.85,3.15)	4.22 (2.28,7.82)	
**Physical activity**				＜0.001
Inactive (N = 1735)	1.00	2.55 (1.40,4.65)	6.24 (3.54,11.03)	
Partially active (N = 433)	1.00	0.87 (0.41,1.88)	0.62 (0.17,2.31)	
Active (N = 372)	1.00	0.98 (0.35,2.72)	2.11 (0.73,6.08)	

Adjusted for age, gender, monthly living expenses, major, smoking, drinking, and physical activity. Of note, variables examined in this table were not adjusted.

Table S5. Stratified analyses for association between external eating and irritable bowel syndrome.

Exposure	External Eating			*p* for Interaction
	Tertile 1	Tertile 2	Tertile 3	
**Gender**				＜0.001
Male (N = 1718)	1.00	2.46 (1.38,4.38)	6.10 (3.62,10.27)	
Female (N = 1021)	1.00	3.13 (1.51,6.47)	4.32 (2.09,8.91)	
**Monthly living expenses (yuan)**				＜0.001
≤1200 (N = 1381)	1.00	2.55 (1.40,4.67)	4.89 (2.79,8.55)	
＞1200 (N = 1358)	1.00	3.52 (1.80,6.88)	6.71 (3.52,12.78)	
**Major**				＜0.001
Clinical Medicine (N = 908)	1.00	2.80 (1.26,6.23)	3.41 (1.55,7.48)	
Others (N = 1831)	1.00	3.20 (1.87,5.47)	6.81 (4.12,11.27)	
**Smoking**				＜0.001
No (N = 1805)	1.00	3.02 (1.75,5.22)	4.74 (2.75,8.15)	
Yes (N = 934)	1.00	2.65 (1.22,5.79)	6.39 (3.19,12.80)	
**Drinking**				＜0.001
No (N = 1519)	1.00	4.42 (2.33,8.38)	6.31 (3.33,11.96)	
Yes (N = 1220)	1.00	1.88 (1.01,3.52)	4.63 (2.64,8.13)	
**Physical activity**				＜0.001
Inactive (N = 1735)	1.00	3.13 (1.80,5.44)	6.69 (4.01,11.16)	
Partially active (N = 433)	1.00	1.73 (0.63,4.74)	3.05 (1.05,8.81)	
Active (N = 372)	1.00	2.53 (0.75,8.58)	2.51 (0.73,8.61)	

Adjusted for age, gender, monthly living expenses, major, smoking, drinking, and physical activity. Of note, variables examined in this table were not adjusted.

Table S6. Stratified analyses for association between restraint eating and irritable bowel syndrome.

Exposure	Restraint Eating			*p* for Interaction
	Tertile 1	Tertile 2	Tertile 3	
**Gender**				**<0.001**
Male (N = 1718)	1.00	4.01 (2.07,7.76)	9.49 (5.08,17.82)	
Female (N = 1021)	1.00	1.62 (0.90,2.93)	2.06 (1.10,3.84)	
**Monthly living expenses (yuan)**				**<0.001**
≤1200 (N = 1381)	1.00	4.13 (2.08,8.23)	8.34 (4.27,16.27)	
>1200 (N = 1358)	1.00	1.97 (1.12,3.50)	3.48 (1.98,6.11)	
**Major**				**<0.001**
Clinical Medicine (N = 908)	1.00	2.21 (1.11,4.42)	2.24 (1.06,4.74)	
Others (N = 1831)	1.00	3.35 (1.90,5.88)	7.62 (4.42,13.12)	
**Smoking**				**<0.001**
No (N = 1805)	1.00	2.63 (1.61,4.29)	3.41 (2.01,5.78)	
Yes (N = 934)	1.00	2.99 (1.17,7.66)	9.11 (3.84,21.59)	
**Drinking**				**<0.001**
No (N = 1519)	1.00	3.20 (1.82,5.61)	4.11 (2.26,7.48)	
Yes (N = 1220)	1.00	2.13 (1.08,4.23)	5.80 (3.07,10.98)	
**Physical activity**				**<0.001**
Inactive (N = 1735)	1.00	2.92 (1.67,5.11)	6.48 (3.82,10.98)	
Partially active (N = 433)	1.00	2.03 (0.86,4.83)	0.78 (0.22,2.84)	
Active (N = 372)	1.00	2.21 (0.63,7.74)	5.10 (1.49,17.44)	

Adjusted for age, gender, monthly living expenses, major, smoking, drinking, and physical activity. Of note, variables examined in this table were not adjusted.
